# Association of Vitamin C Supplementation and Genetic Susceptibility with Multiple Sclerosis Risk: A Prospective Population-Based Cohort Study

**DOI:** 10.3390/nu18142367

**Published:** 2026-07-20

**Authors:** Andrea Nova, Teresa Fazia, Giovanni Di Caprio, Alice Cinquepalmi, Simone Perna, Mariangela Rondanelli, Luisa Bernardinelli

**Affiliations:** 1 Department of Brain and Behavioral Sciences, University of Pavia, 27100 Pavia, Italy; teresa.fazia@unipv.it (T.F.); giovanni.dicaprio01@universitadipavia.it (G.D.C.); alice.cinquepalmi01@universitadipavia.it (A.C.); luisa.bernardinelli@unipv.it (L.B.); 2 Division of Human Nutrition, Department of Food, Environmental and Nutritional Sciences (DeFENS), University of Milan, 20122 Milan, Italy; simone.perna@unimi.it; 3 Department of Public Health, Experimental and Forensic Medicine, University of Pavia, 27100 Pavia, Italy; mariangela.rondanelli@unipv.it

**Keywords:** vitamin C, supplementation, multiple sclerosis, genetic risk, UK Biobank, propensity score

## Abstract

Background/Objectives: Multiple sclerosis (MS) is a chronic immune-mediated neurological disorder for which few modifiable risk factors are established. Vitamin C, due to its antioxidant and neuroprotective properties, has been hypothesized to reduce MS risk, but epidemiological evidence remains inconsistent. Methods: We conducted a prospective cohort study using UK Biobank data, including 486,908 adults aged 37–70 years free of neurological disease. Regular vitamin C supplementation was self-reported at recruitment. Incident MS cases were identified during a median follow-up of 13.5 years. Propensity score weighting was used to balance a wide range of demographic, lifestyle, and health-related confounders. Weighted Cox proportional hazards models were used to estimate hazard ratios (HRs). Effect modification by polygenic risk score (PRS) for MS was assessed, and multiple sensitivity analyses were performed. Results: During follow-up, 452 participants were diagnosed with MS. Vitamin C supplementation was reported by 8.8% of participants (missingness = 1.5%) and was associated with a lower risk of incident MS (HR = 0.51, [95%CI: 0.32; 0.82], *p* = 0.004). The estimate was consistent across multiple sensitivity analyses. The association varied according to genetic susceptibility, with significant nonlinear multiplicative interaction (*p* = 0.004) and additive interaction (*p* < 0.001). Specifically, significant associations were observed only among those with average or high MS-PRS. Conclusions: Vitamin C supplementation was associated with a lower risk of incident MS, with the association varying across levels of genetic susceptibility. Although residual confounding cannot be excluded, sensitivity analyses did not identify similar associations for overall supplement use or multivitamin use, providing some reassurance against a generalized healthy-user effect. Replication in independent cohorts is warranted.

## 1. Introduction

Multiple Sclerosis (MS) is a chronic immune-mediated demyelinating disorder of the central nervous system and a leading cause of non-traumatic neurological disability in young and middle-aged adults [1]. Recent evidence suggests that the incidence and prevalence of MS have increased over time, with a shift toward older age at diagnosis, potentially reflecting changes in environmental exposures and improved disease diagnosis [2,3,4]. These trends highlight the need to identify modifiable factors that could lower the risk of disease onset and its burden.

To date, only a few have been consistently established, namely smoking, vitamin D deficiency, and obesity [5,6]. Given the role of oxidative stress and neuroinflammation in MS pathophysiology [7,8], vitamin C (ascorbic acid) has been hypothesized to influence MS risk given its antioxidant and neuroprotective properties [7,9]. Notably, a recent experimental study using an animal model of MS suggested that vitamin C, in combination with vitamin A, promotes resolution of neuroinflammation and supports remyelination [10,11].

However, epidemiological evidence linking vitamin C to MS risk remains limited and inconsistent. While a case-control study reported lower MS risk associated with higher vitamin C intake [12], a prospective cohort study found no significant association for either dietary vitamin C intake or supplementation [13]. Similarly, a Mendelian randomization study did not provide statistically significant evidence for a causal association between genetically predicted plasma vitamin C concentrations and MS risk [14], although the point estimate was directionally consistent with a modest protective association. Differences across these studies may reflect variations in exposure assessment (dietary intake, supplementation, or genetically predicted circulating levels), study design, statistical power, and confounding control. Importantly, genetically predicted circulating vitamin C concentrations represent a different exposure construct from supplementation and may not fully capture the biological consequences of supplement use [15]. Moreover, previous studies have not evaluated whether the association between vitamin C supplementation and MS risk may differ according to underlying genetic susceptibility. These discrepancies highlight the need for further prospective studies using large-scale data.

To address these gaps, we used the UK Biobank’s large population-based cohort of adults free of neurological disease at baseline and followed-up for more than ten years. The prospective nature of the study combined with the large amount of information, including data on lifestyle, health status, and other concurrent vitamins and mineral supplement use, allowed implementation of a propensity score (PS) framework with extensive confounding adjustment. As a secondary analysis, we also examined whether the association differed according to genetic susceptibility measured through a polygenic risk score for MS (MS-PRS). By combining information obtained in a real-world setting, this study aims to clarify whether vitamin C supplementation is associated with incident MS risk and whether this association is modified by genetic susceptibility to MS.

## 2. Materials and Methods

This study is an observational study conducted according to the Strengthening the Reporting of Observational Studies in Epidemiology (STROBE) reporting guideline for observational studies.

### 2.1. Data

The UK Biobank [16] (N = 501,924) offers a comprehensive range of health-related phenotypes and genetic data collected from individuals recruited between 2006 and 2010 and aged 37 to 70. Individuals were then followed over time, recording any new clinical diagnosis.

#### 2.1.1. Multiple Sclerosis Definition

Participants with prevalent MS at baseline were identified where G35 diagnosis code, defined according to the International Classification of Diseases 10th Revision (ICD-10), was present before recruitment from hospital records, primary care records, death registries, and physician-confirmed self-reported diagnoses. These individuals were excluded from the cohort. Incident MS was subsequently ascertained exclusively through hospital admission records with an ICD-10 diagnosis of G35 occurring after recruitment. To better approximate the timing of disease onset, among participants who developed incident MS, the diagnosis date was backdated when an earlier record of a neurological diagnosis compatible with a first MS symptom (ICD-10: G36-G37, H46-H47, or R90). These auxiliary codes were used solely to refine the timing of confirmed MS diagnoses and were not used to define additional MS cases.

#### 2.1.2. Target Population

The study population comprised middle-aged and older adults free from prior diagnoses of MS, demyelinating events, and neurodegenerative diseases at baseline. We excluded individuals with a diagnosis of MS (G35) prior to recruitment (N = 2007) or within 2 years thereafter (N = 27), to minimize the inclusion of prevalent cases with delayed recording. To further reduce the likelihood of including individuals with undiagnosed MS, we excluded participants with a history of demyelination or neurological conditions, such as optic neuritis, encephalitis and spinal cord disease (N = 13,174) (see Supplementary Material for a complete list). We also removed individuals with prevalent Parkinson’s and Alzheimer’s disease, for a total of 15,208 individuals removed. A flow-chart reporting these steps was reported in Figure S1.

#### 2.1.3. Vitamin C Supplementation

Regular vitamin C supplementation at recruitment was ascertained from UK Biobank field 6155, in which participants indicated “Vitamin C” in response to the question “Do you regularly take any of the following vitamin and mineral supplements?”. We additionally considered field 20003, in which participants self-reported regularly taking a “vitamin c product” or “ascorbic acid product” (see Table S2 for number of individuals across the two sources). No information was available regarding dosage, formulation, duration of use, or changes in supplementation during follow-up. Products combining vitamin C with other vitamins were classified instead as multivitamins. Therefore, this exposure primarily captures standalone vitamin C supplementation, rather than low-dose vitamin C contained within multivitamin preparations.

#### 2.1.4. Confounders

We considered several variables as potential confounders for the relationship between vitamin C supplementation and MS diagnosis. These include (i) demographics, i.e., sex, age at recruitment, ethnicity (white vs. non-white), country of birth (UK/Ireland, Other), education level (College, High School, None), Townsend deprivation index, average income (<18,000£, 18,000 to 30,999£, 31,000 to 51,999£, 52,000 to 100,000£, >100,000£), (ii) MS risk factors. i.e., family history of nervous system disorders, history of infectious mononucleosis diagnosis (ICD-10: B27), self-reported body size at age 10 (Plumper vs. Average or Thinner), body mass index, smoking habits (Never, Previous, Current), vitamin D levels in the blood, (iii) lifestyle factors, i.e., alcohol consumption (Never, Previous, Current), physical activity (Never, Moderate, High), average hours spent outdoor per week, adherence to selected Eatwell Guide dietary recommendations [17], i.e., ≥5 portions/day of fruits or vegetables, ≥2 portions/week of oily fish, ≤3 portions/week of unprocessed meat, ≤1 portion/week of processed meat, and daily cups of caffeinated coffee, (iv) health status, i.e., long-standing illness (yes vs. no), self-perceived health rating (poor, fair/good, excellent), illness or injury in last two years (yes vs. no), total number of self-reported medications (excluding supplements), v) intake of other vitamin and mineral supplements: Vitamin A, Vitamin B, Vitamin B9 (folic acid), Vitamin E, multivitamins, fish oil/cod liver oil, glucosamine, zinc, calcium, iron, selenium. The corresponding UK Biobank data fields were reported in Table S1.

### 2.2. Statistical Analysis

#### 2.2.1. Imputation

Descriptive statistics were computed for all analyzed variables at the time of recruitment, comparing individuals with and without vitamin C supplementation. Missing data for vitamin C supplementation (1.5%) and confounders were handled using multiple imputations by chained equations [18]. We imputed ten different datasets and pooled results using Rubin’s rule. All statistical analyses were performed in R (RStudio version 2020.05.0). A template’s R script is provided in the Supplementary Material.

#### 2.2.2. Study Design

Our analysis was based on prospective cohort study design. Specifically, we considered the observation time of each individual starting from the date of recruitment and continuing up to the earliest date among MS diagnosis, death, loss to follow-up, or the study’s end (31 December 2022).

#### 2.2.3. Propensity Score and Weighted Cox Model

In the main analysis, we investigated the association between regular vitamin C supplementation and incident MS. To address confounding, we estimated PSs representing the probability of vitamin C supplementation conditional on baseline confounders. The PS was derived using a logistic regression model where vitamin C supplementation was the dependent variable, while all previously listed confounders were the independent variables. Continuous variables were modeled a priori using splines with 3 knots to allow a flexible yet parsimonious adjustment for potential non-linear confounding in the PS model, while avoiding unnecessary overfitting.

Inverse probability of treatment weights (IPTWs) were calculated as the inverse of the PS for exposed individuals and the inverse of one minus the PS for unexposed individuals (R package ipw) [19]. We evaluated the presence of extreme IPTWs (i.e., IPTWs > 10), and in case we truncated at the 1st and 99th percentiles. Covariate balance before and after weighting was assessed using absolute standardized mean differences (SMDs), with values < 0.1 considered indicative of adequate balance (R package cobalt). The distribution of PSs was visually inspected to evaluate overlap and identify potential violations of the positivity assumption [20]. Weighted Cox proportional hazards (PHs) models were then fitted to estimate hazard ratios (HRs) and 95% confidence intervals (CIs) for the association between vitamin C supplementation and MS risk. Inspection of Schoenfeld residuals was conducted to assess the PH assumption. We also exploratively performed subgroup analysis by sex and age (categorized as <60 and ≥60 years).

#### 2.2.4. Sensitivity Analyses

First, a sensitivity analysis was conducted to assess associations’ robustness by calculating the E-value (R package EValue) [21], defined as the minimum strength of association, on the risk-ratio scale, that an unmeasured confounder would need to have with both vitamin C supplementation and MS risk, conditional on the measured confounders, to remove any significant association.

Second, we evaluated the robustness of the results by (i) excluding individuals with missing information on vitamin C supplementation, (ii) extending the exclusion window for MS diagnoses occurring shortly after recruitment from 2 years to 5 years to assess whether our results were influenced by reverse causality, and (iii) by not excluding individuals with history of demyelination, neurological conditions and neurodegenerative diseases at the time of recruitment to assess the potential impact of this selection.

Third, to further evaluate whether the observed association could be explained by a generalized supplement-use behavior rather than a vitamin C-specific association, we performed additional supplement specificity analyses. We examined the association between overall supplement use and incident MS, defined as regular use of any vitamin or mineral supplement, compared with no supplement use. We additionally examined multivitamin use as another marker of general health-conscious behavior. These analyses were performed using the same IPTWs and Cox proportional hazards modelling framework as the primary analysis.

Fourth, we performed negative control outcome analyses to evaluate the potential impact of residual confounding [22]. Negative control outcomes are outcomes for which a causal association with the exposure is considered unlikely, and any observed association may suggest the presence of systematic bias or uncontrolled confounding. We considered pedestrian and transport accidents (ICD-10 codes V00-V99) and acute appendicitis (ICD-10 code K35) as negative control outcomes with no established biological relationship with vitamin C supplementation. For each negative control outcome, we applied the same IPTWs and Cox proportional hazards modelling approach used in the primary analysis, with follow-up calculated from recruitment until the occurrence of the outcome, death, loss to follow-up, or end of follow-up.

#### 2.2.5. Interactions with MS Polygenic Risk Score

In the secondary analysis, we investigated whether the association between vitamin C supplementation and incident MS varied according to genetic susceptibility. To this aim, we included in the main model an interaction term between vitamin C supplementation and the MS-PRS. MS-PRS was standardized so that the mean was equal to 0 and the standard deviation (SD) equal to 1. To adjust for any confounding between MS-PRS and MS, we added as covariates sex, age at recruitment, country of birth, ethnicity, and the first ten genetic principal components, as provided by the UK Biobank. IPTWs were instead used to adjust for vitamin C supplementation-MS confounding.

MS-PRS was modeled using restricted cubic splines with three knots to allow for potential non-linear associations with MS risk and to avoid imposing a priori assumptions of linearity. Wald tests were used to assess the contribution of the non-linear spline components for both the non-linear effect of MS-PRS and its interaction with vitamin C supplementation. HRs across the MS-PRS distribution were estimated using counterfactual contrasts derived from the fitted model and used to visualize the exposure-specific association as a function of genetic risk. To assess interaction on the additive scale, we estimated the relative excess risk due to interaction (RERI) [23]. We used the formula RERI = HR+/+ − HR+/− − HR−/+ + 1, where + denotes the presence of the exposure, − denotes the lack of exposure, and HR denotes the Hazard Ratio within a specific combination estimated relative to the lack of both exposures (−/−). Vitamin C supplementation use was used as the risk exposure of interest (+) so that the absence of supplementation represented the reference exposure category (−), while for MS-PRS we considered low (−1 SD) (−) and high (+1 SD) (+) MS-PRS values. This parameterization yields a positive/negative RERI when the joint risk effect of higher genetic susceptibility and vitamin C supplementation is above/below the sum of their individual effects [24]. RERI was estimated by comparing counterfactual contrasts based on the fitted model. 95% Confidence intervals for the RERI and HRs were obtained using the bias-corrected and accelerated (BCa), and two-sided *p*-values were obtained from bootstrap confidence intervals (R package boot.pval).

## 3. Results

### 3.1. Descriptive Statistics

We analyzed data from 486,908 individuals aged 37–70 (mean: 57 years), most of whom were born in the UK and of white ethnicity Over a median observation time of 13.45 years (IQR: 13.06; 14.53), 452 participants were diagnosed with MS, at a mean of 7.69 years (IQR: 4.86; 10.43) after recruitment. Vitamin C supplementation was reported by 42,728 individuals (8.8%), and information was missing for 7076 (1.5%). Of the 452 incident MS cases, 427 occurred in non-users (0.98‰) and 19 in users of vitamin C supplementation (0.44‰), with 6 among participants whose exposure was missing (0.85‰).

**Figure 1:**
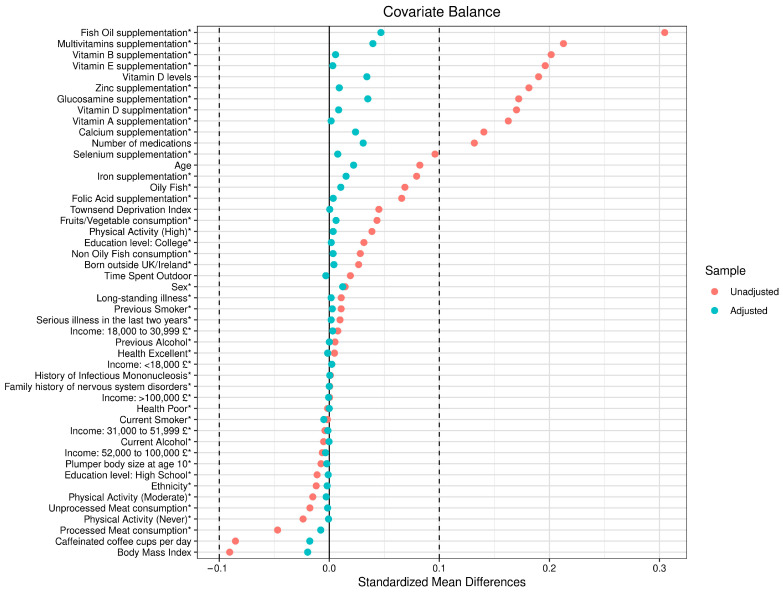
Standardized mean differences, for each variable included in the propensity score calculation, before and after adjustment using inverse probability of treatment weights. Absolute values below 0.1 indicated adequate balance among users and non-users of vitamin C supplementation. The * denotes binary variables.

Descriptive statistics for each potential predictor at the time of recruitment, stratified by supplementation status, were reported in Table S3. Vitamin C users were substantially more likely to use other vitamins/mineral supplements, take more medications, and have higher vitamin D levels. This can be observed by looking at the SMDs before using IPTWs in Figure 1 (red dots), where these variables were severely unbalanced between exposed and non-exposed (|SMD| > 0.1).

These differences may reflect greater health-conscious behaviors among vitamin C users. Moreover, even with smaller differences, individuals using vitamin C supplementation had a higher education level and lower socioeconomic deprivation, were more physically active, and had a higher diet quality.

### 3.2. Association Between Vitamin C Supplementation and MS Risk

The following analyses were implemented within each imputed dataset and then pooled using Rubin’s rule.

**Figure 2:**
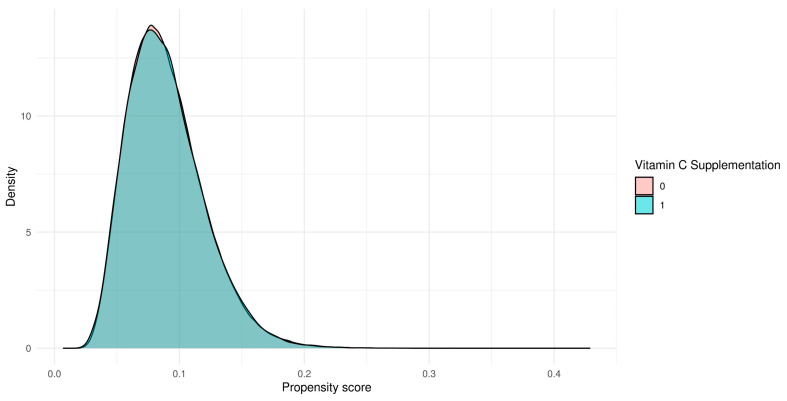
Propensity score distributions overlap among users and non-users of vitamin C supplementation.

We first calculated the IPTWs, which had a minimum value equal to 0.21 and maximum value of 3.93. We therefore did not perform IPTWs truncation. The resulting effective sample size was 481,811 (Non exposed: 442,801.8, Exposed: 39,059.02), indicating a small reduction relative to the original cohort size. Covariates balance between exposed and non-exposed before and after IPTWs-weighting was depicted in Figure 1. Absolute adjusted SMD was below 0.1 for all measured confounders, therefore providing evidence of achieved covariate balance. Moreover, PSs distributions showed substantial overlap between exposure and non-exposed, providing evidence against violations of the positivity assumption (see Figure 2).

**Figure 3:**
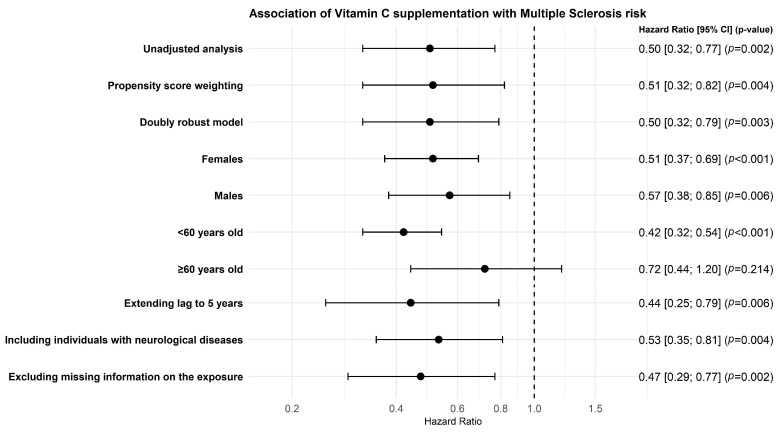
Association of Vitamin C supplementation and Multiple Sclerosis risk in the main analysis, subgroup analyses, and sensitivity analysis.

All results obtained in the following analyses were depicted in Figure 3.

In the weighted Cox model, vitamin C supplementation was associated with a 49% lower risk of MS relative to non-use (HR = 0.51, [95% CI: 0.32; 0.82], *p* = 0.004). We found no evidence of deviation from the PH assumption (*p* = 0.750) (see Figure S2 for Schoenfeld residuals plot). Results did not change using a doubly robust model, obtained by fitting a weighted Cox model additionally adjusted for all baseline covariates included in the PS model, as well as performing an unadjusted analysis (see Figure 3).

Exploratory subgroup analysis by sex and age group (<60 and ≥60) showed very similar associations with no significant interaction (*p* = 0.806 and *p* = 0.238), even though a stronger association was observed in younger individuals (see Figure 3).

### 3.3. Interaction with MS Polygenic Risk Score

In the secondary analysis, we evaluated whether the association between vitamin C supplementation and incident MS varied according to genetic susceptibility.

The descriptive distribution of individuals, incident MS cases, and crude MS incidence according to vitamin C supplementation status and MS-PRS quartiles was reported in Table S4. MS-PRS was not associated with vitamin C supplementation use (*p* = 0.813 using a chi-square test).

**Figure 4:**
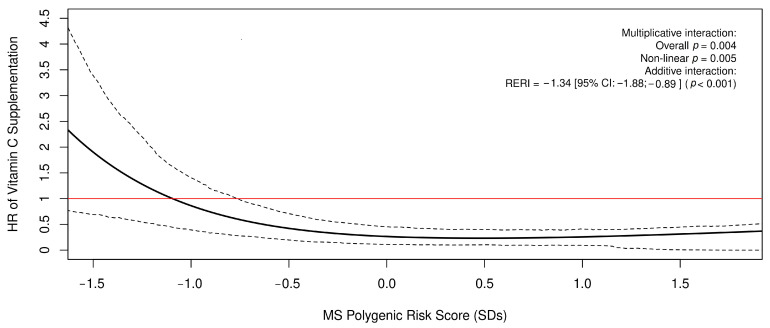
Association, in terms of Hazard Ratio, between vitamin C supplementation use and Multiple Sclerosis risk given the distribution of standardized Multiple Sclerosis (MS) Polygenic Risk Score from the 5th to the 95th percentiles. A negative RERI here indicates that vitamin C supplementation use combined with higher genetic susceptibility is associated with a lowered MS risk due to their interaction. Red horizontal line denotes the null association (Hazard Ratio = 1). Black line denotes the estimated Hazard Ratio. Dotted lines denote the 95% confidence intervals. SD = Standard Deviation, RERI = Relative Excess Risk due to Interaction, HR = Hazard Ratio.

We modeled the MS-PRS as a continuous variable using restricted cubic splines with three knots. MS-PRS showed a significant overall association with incident MS (*p* < 0.001), with evidence of non-linearity (*p* = 0.014). Importantly, the multiplicative interaction between vitamin C supplementation and MS-PRS was statistically significant (*p* = 0.004), indicating that the association between vitamin C supplementation and MS risk differed across the distribution of genetic susceptibility. The interaction was driven by a significant non-linear component (*p* = 0.005), suggesting that the magnitude of the association varied non-linearly with increasing MS-PRS. HRs for vitamin C supplementation use across the MS-PRS distribution are shown in Figure 4.

The plot showed that non-significant associations were observed among individuals with low MS-PRS values (below approximately 0.8 SD from the population mean), whereas significant inverse associations were observed among individuals with average to high MS-PRS values. Specifically, for MS-PRS values equal to −1 SD (low genetic risk), 0 (average genetic risk), and +1 SD (high genetic risk), the estimated HRs associated with vitamin C supplementation were 0.86 (95% CI: 0.39; 1.41), 0.27 (95% CI: 0.11; 0.45), and 0.26 (95% CI: 0.09; 0.41), respectively. This pattern was consistent with the significant nonlinear interaction between vitamin C supplementation and MS-PRS.

On the additive scale, using MS-PRS values of +1 SD and −1 SD to define the genetic risk contrast, the estimated RERI was −1.34 (95% CI: −1.88; −0.89; *p* < 0.001), indicating a significant negative additive interaction between vitamin C supplementation use and higher genetic susceptibility.

### 3.4. Sensitivity Analyses

We then assessed how vulnerable the association was to unmeasured confounding. The point estimate (HR = 0.51) yielded an E-value of 3.33, meaning that an unmeasured confounder would need to be associated with both vitamin C supplementation and MS risk by a risk ratio of at least 3.33 each, beyond the measured confounders, to fully explain the association away. Considering the upper confidence limit (0.82), we obtained an E-value equal to 1.74, i.e., the strength of unmeasured confounding that would be required to render the association non-significant.

Exclusion of individuals with missing information on vitamin C supplementation, extension of the lag-window for MS cases exclusion from 2 to 5 years after the recruitment (127 MS cases excluded), and inclusion of the previously excluded 13,174 individuals with history of neurological and neurodegenerative diseases did not affect the main findings (see Figure 3).

Additive interactions remained statistically significant and of similar magnitude across all three scenarios (Table S5). Evidence for multiplicative interaction was likewise consistent, although it no longer reached statistical significance when a 5-year lag was applied (*p* = 0.078), likely reflecting the reduced number of incident MS cases available for analysis.

In additional supplement specificity analyses, both overall supplement use (HR = 1.06, 95% CI: 0.87; 1.29, *p* = 0.554) and multivitamin use were not associated with a reduced risk of MS (HR = 1.28, 95% CI: 0.98; 1.65, *p* = 0.067). These findings suggest that the inverse association observed for vitamin C supplementation was not observed in general supplement users.

Lastly, we performed negative control outcome analyses, where incident pedestrian/transport accidents (N = 5794) and appendicitis (N = 4842) were considered as outcomes in the weighted Cox model. Vitamin C supplementation was not associated with the risk of transport and pedestrian accidents (HR = 1.04, 95% CI: 0.94; 1.15, *p* = 0.432) and acute appendicitis (HR = 1.03, 95% CI: 0.89; 1.19, *p* = 0.670). These findings provide further reassurance against major systematic bias but cannot completely exclude residual confounding.

## 4. Discussion

In this prospective cohort of nearly half a million UK adults aged 37–70, regular vitamin C supplementation, defined as the use of standalone vitamin C products, was associated with a lower risk of incident MS (HR = 0.51, 95% CI: 0.32; 0.82) after extensive confounding adjustment, and this association remained consistent across multiple sensitivity analyses.

Notably, this association was not homogeneous across the cohort, but varied according to genetic susceptibility to MS. To our knowledge, such a gene-environment interaction pattern has not previously been reported for vitamin C supplementation. Specifically, the inverse association was significant and of similar magnitude among individuals with average and higher MS genetic susceptibility, whereas it was attenuated toward the null and no longer statistically significant among those with lower MS-PRS values. The reasons underlying this pattern remain uncertain. One possible explanation is that individuals with lower genetic susceptibility have a lower baseline risk of MS, leaving less opportunity for an observable association with vitamin C supplementation, whereas any potential association may be more readily detectable among individuals at higher underlying risk. However, given the limited number of MS cases among vitamin C users, this interaction finding should be interpreted with caution. Consistent with this limitation, when a more stringent 5-year lag period was applied in sensitivity analyses, the evidence for multiplicative interaction no longer reached statistical significance, although the additive interaction remained similar in magnitude and statistically significant.

The biological mechanisms linking vitamin C supplementation to MS risk remain uncertain. Vitamin C has antioxidant and immunomodulatory properties and has been implicated in processes relevant to MS pathophysiology, including oxidative stress regulation [7,8], immune responses, and myelin biology [25,26,27]. Experimental evidence in animal models suggests that vitamin C may promote resolution of neuroinflammation and support remyelination [10,11,28]. However, the extent to which oral supplementation influences vitamin C concentrations within the central nervous system in humans is unclear. Vitamin C transport across the blood-brain barrier is tightly regulated by specific transport systems, including Sodium Vitamin C co-transporter 2 [29], and circulating vitamin C levels may not directly reflect central nervous system availability. Therefore, our findings should not be interpreted as evidence that typical supplementation doses necessarily produce clinically meaningful increases in brain vitamin C concentrations. Rather, the observed association provides epidemiological support for further investigation of the biological pathways through which vitamin C availability may influence MS susceptibility.

These findings are consistent with a previous case-control study reporting a lower MS risk associated with higher vitamin C intake [12], but differ from both a prospective cohort study and a Mendelian randomization analysis that did not identify significant associations [13,14]. Overall, the available epidemiological evidence therefore remains inconsistent. Such discrepancies may reflect differences in vitamin C assessment (dietary intake, standalone supplementation, or genetically predicted plasma levels), exposure timing, study design, statistical power, and confounding structures. In particular, while our study evaluated regular use of standalone vitamin C supplements, the Mendelian randomization study by Peng et al. [14] estimated the effect of approximately one SD higher genetically predicted circulating vitamin C concentrations (~20 μmol/L). Importantly, although their estimate was not statistically significant (Odds Ratio: 0.88, [95% CI: 0.65; 1.18]), the point estimate remained directionally consistent with a protective association. Furthermore, lifelong genetically determined differences in circulating vitamin C concentrations may not fully capture the effects of supplementation, including differences in exposure timing, magnitude of change, bioavailability, or intracellular vitamin C availability [14]. Therefore, the two approaches address different exposure contrasts, particularly regarding timing and magnitude of vitamin C exposure, and are not expected to yield directly comparable effect estimates. Nevertheless, residual confounding remains a plausible explanation for the stronger association observed in our study and cannot be excluded. Replication in independent prospective cohorts and complementary approaches will be then important to clarify the relationship between vitamin C and MS risk.

Strengths of our study include the availability of a large prospective cohort with long follow-up and detailed individual-level information on lifestyle, health-related factors, and use of other supplements, enabling extensive confounding adjustment through propensity-score methods. Furthermore, the availability of an MS-PRS allowed investigation of potential effect modification by genetic susceptibility, an analysis that previous studies were not designed to address.

Several limitations should be considered. First, the lack of validation in an external cohort limits the robustness of the result. Second, given the observational design, residual confounding cannot be fully excluded, and we therefore do not advocate vitamin C supplementation for MS prevention based on these findings alone. One potential source of residual confounding is that individuals using vitamin C supplements may engage in healthier behaviors not fully captured by the available data. To mitigate this possibility, we adjusted for a broad range of lifestyle, health-related, and socioeconomic factors, including use of other supplements and medications, which may reasonably serve as proxies for health-conscious behaviors. Moreover, additional sensitivity analyses examining any supplement use and multivitamin use did not identify similar inverse associations with incident MS, providing some reassurance that the observed association was not solely explained by a generalized supplement-user phenotype. Under the assumptions of the E-value framework, a relatively strong unmeasured confounder associated with both vitamin C supplementation and MS risk would still be required to fully explain the observed association. Third, regular vitamin C supplementation was assessed only at baseline through self-report, and information on dose, formulation, duration of use, and changes in supplementation habits during follow-up was unavailable. Therefore, dose-response relationships or changes in exposure over time could not be captured. Moreover, information on dietary vitamin C intake was unavailable. Although we adjusted for fruit and vegetable consumption as proxy indicators of dietary patterns, we could not directly account for total vitamin C intake from food sources. Fourth, the cohort consisted predominantly of individuals of European ancestry and healthier than the general population, which may limit generalizability. Fifth, despite the large cohort size and long follow-up, the number of incident MS cases among vitamin C users was limited. Therefore, while this study provides a unique opportunity to investigate this association in a large and deeply characterized cohort, the precision of the exposure-specific estimate remains constrained by the number of observed events. The confidence interval (0.32; 0.82) indicates uncertainty in the precise magnitude of the association, with the upper confidence limit still corresponding to an estimated 18% lower risk of MS. Lastly, MS has a potentially prolonged prodromal phase during which non-specific symptoms could influence health-related behaviors [30]. Therefore, we cannot completely exclude the possibility that preclinical disease influenced supplement use before diagnosis, although results were consistent applying a 5-year lag period, providing some reassurance against reverse causation, although longer prodromal effects cannot be completely excluded.

From a clinical and public-health perspective, vitamin C supplementation represents an interesting candidate for further investigation because it is a widely available and relatively low-cost exposure. However, given the observational nature of this study and the possibility of residual confounding, our findings should not be interpreted as evidence supporting vitamin C supplementation for MS prevention. Rather, they provide a rationale for further research, including replication in independent cohorts and, ultimately, intervention studies if consistent evidence accumulates. Our observation that the association may vary according to genetic susceptibility is hypothesis-generating and suggests that future prevention studies could consider whether individuals with different levels of genetic risk respond differently to modifiable exposures. However, the current evidence is insufficient to inform genotype-based prevention strategies or clinical recommendations.

## 5. Conclusions

In conclusion, in the large UK Biobank prospective cohort, regular vitamin C supplementation was associated with a lower risk of incident MS, particularly among individuals with average to high genetic susceptibility. Although residual confounding cannot be excluded, these findings provide a rationale for further investigation in independent cohorts, functional studies, and, ultimately, randomized intervention trials to determine whether vitamin C supplementation has a causal role in MS prevention and whether this differs according to genetic susceptibility.

## Data Availability

No new data were created or analyzed in this study. Data sharing is not applicable to this article. Researchers may obtain access to the data used in this study upon reasonable request to the UK Biobank study team (https://www.ukbiobank.ac.uk/).

## Supplementary Materials

The following supporting information can be downloaded at https://www.mdpi.com/article/10.3390/nu18142367/s1, Table S1: Description of the variables with corresponding UK Biobank data fields; Table S2: Vitamin C supplementation users distribution across two UK Biobank data fields 6155 and 20003; Table S3: Descriptive statistics stratified by vitamin C supplementation users and non-users; Table S4: Number of Multiple Sclerosis cases and raw incidence stratified by vitamin C supplementation use and polygenic risk score quartiles; Table S5: Interaction between vitamin C supplementation and Multiple Sclerosis Polygenic Risk Score in the sensitivity analyses; Figure S1: Flowchart describing the steps applied to obtain the analyzed dataset; Figure S2: Plot of Schoenfeld’s residuals from the main Cox model analyzing the association between vitamin C supplementation and Multiple Sclerosis risk; Supplemental Material: Neurological conditions excluded at baseline; R code template; STROBE checklist: STROBE Statement—checklist of items that should be included in reports of observational studies.

## Abbreviations

The following abbreviations are used in this manuscript:

CI	Confidence Interval
HR	Hazard Ratio
ICD	International Classification of Diseases
IPTW	Inverse probability of treatment weight
MS	Multiple Sclerosis
PH	Proportional Hazard
PRS	Polygenic Risk Scores
PS	Propensity Score
RERI	Relative Excess Risk due to Interaction
SD	Standard Deviation
SMD	Standardized Mean Difference
