# Supplementary material for: Association of Vitamin C Supplementation and Genetic Susceptibility with Multiple Sclerosis Risk: A Prospective Population-Based Cohort Study
Source: Nutrients. 2026 Jul 20;18(14):2367. doi: 10.3390/nu18142367 (PMC13414532; doi:10.3390/nu18142367)
Supplement: Supplementary file 1 [file nutrients-18-02367-s001.zip › Supplementary Material.pdf]

Supplementary Material for "Association of Vitamin C Supplementation and Genetic Susceptibility with Multiple Sclerosis Risk: A Prospective Population-Based Cohort Study"

Neurological conditions excluded at baseline

To further reduce the likelihood of including individuals with undiagnosed MS, we excluded participants with a history of demyelination or neurological conditions. These included demyelinating diseases in the CNS (G36-G37) (N=11), optic neuritis (H46-H47) (N=762), encephalitis (G04-G05) (N=547), systemic atrophies in the CNS (G10-G14) (N=258), cranial nerve disorders (G50-G53) (N=4044), infantile cerebral palsy (G80) (N=234), disorders of autonomic nervous system (G90) (N=252), hydrocephalus (G91) (N=222), toxic encephalopathy (G92) (N=3), brain disorders (G93-G94, excluding post-viral fatigue G93.3) (N=3132), spinal cord disease (G95) (N=798), and other disorders of the nervous system (G96-G99) (N=2811). We further removed individuals with prevalent neurodegenerative diseases, i.e., Parkinson's disease (G20-G23) (N=957) and Alzheimer's disease (G30-G32) (N=185). We also considered abnormal findings on diagnostic imaging of CNS (R90) dementia (F00-F03), but none had these prior conditions at the time of recruitment.
