# Supplementary figures and images for: Association of Vitamin C Supplementation and Genetic Susceptibility with Multiple Sclerosis Risk: A Prospective Population-Based Cohort Study
Source: Nutrients. 2026 Jul 20;18(14):2367. doi: 10.3390/nu18142367 (PMC13414532; doi:10.3390/nu18142367)

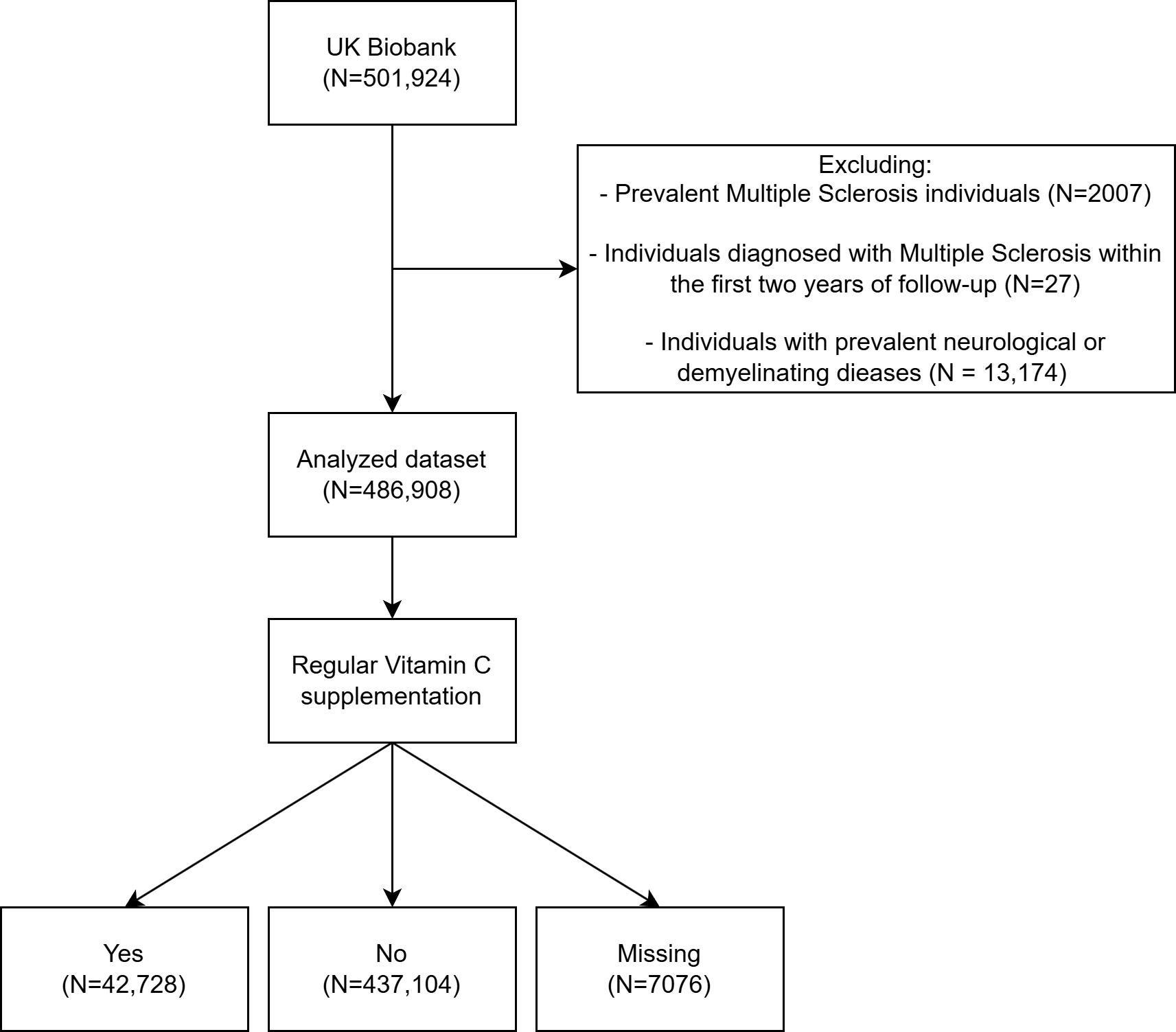

Supplement: Supplementary file 1 [file nutrients-18-02367-s001.zip › Figure S1.png]

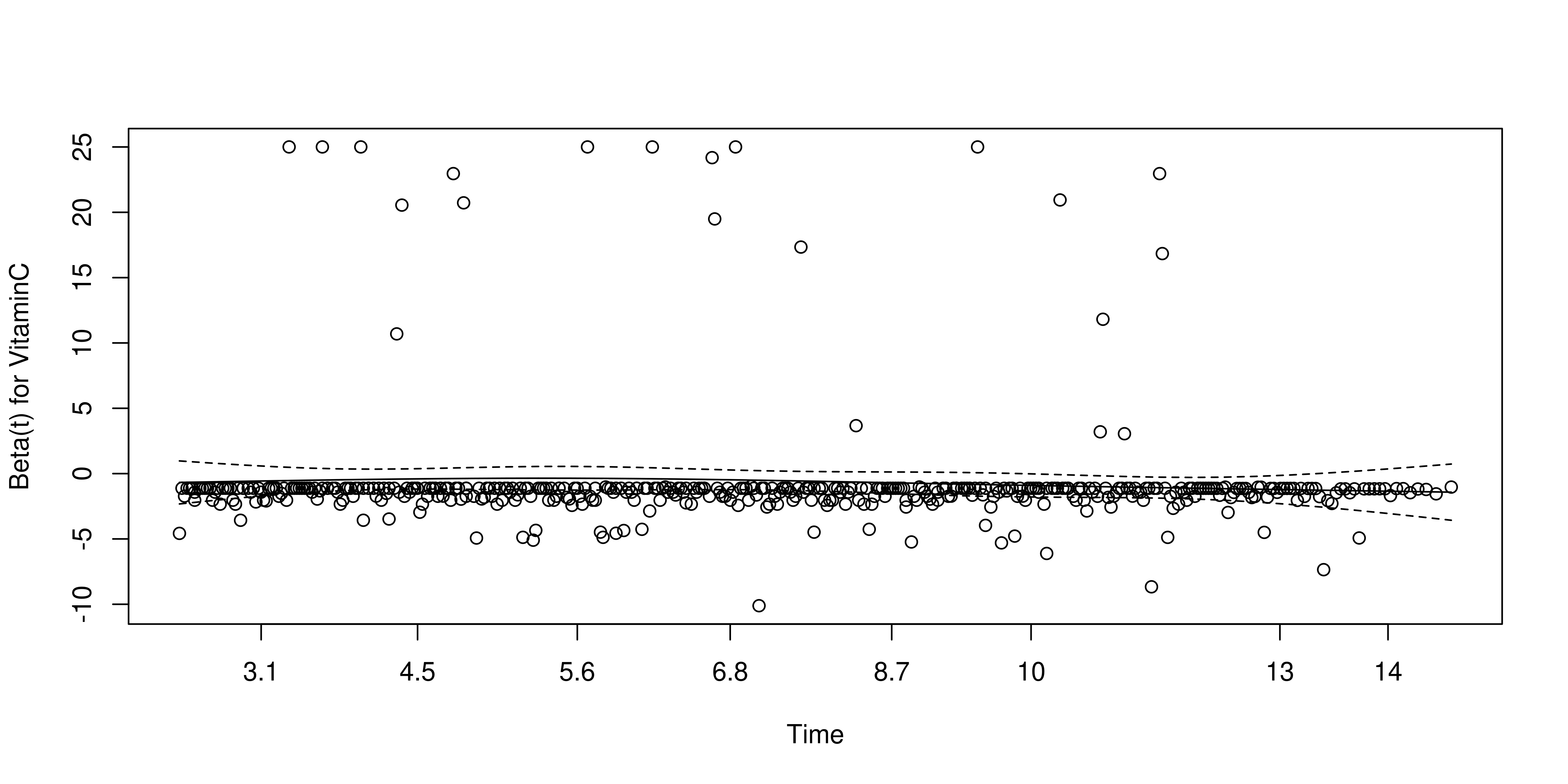

Supplement: Supplementary file 1 [file nutrients-18-02367-s001.zip › Figure S2.tif]
